# VarSim: a high-fidelity simulation and validation framework for high-throughput genome sequencing with cancer applications

**DOI:** 10.1093/bioinformatics/btu828

**Published:** 2014-12-17

**Authors:** John C. Mu, Marghoob Mohiyuddin, Jian Li, Narges Bani Asadi, Mark B. Gerstein, Alexej Abyzov, Wing H. Wong, Hugo Y.K. Lam

**Affiliations:** ^1^Department of Electrical Engineering, Stanford University, Stanford, CA 94035, USA, ^2^Department of Bioinformatics, Bina Technologies, Redwood City, CA 94065, USA, ^3^Program in Computational Biology and Bioinformatics, Yale University, New Haven, CT 06520, USA, ^4^Mayo Clinics, Department of Health Sciences Research, Rochester, MN 55902, USA, ^5^Department of Statistics, Stanford University, Stanford, CA 94035, USA and ^6^Department of Health Research and Policy, Stanford University, Stanford, CA 94035, USA

## Abstract

**Summary:** VarSim is a framework for assessing alignment and variant calling accuracy in high-throughput genome sequencing through simulation or real data. In contrast to simulating a random mutation spectrum, it synthesizes diploid genomes with germline and somatic mutations based on a realistic model. This model leverages information such as previously reported mutations to make the synthetic genomes biologically relevant. VarSim simulates and validates a wide range of variants, including single nucleotide variants, small indels and large structural variants. It is an automated, comprehensive compute framework supporting parallel computation and multiple read simulators. Furthermore, we developed a novel map data structure to validate read alignments, a strategy to compare variants binned in size ranges and a lightweight, interactive, graphical report to visualize validation results with detailed statistics. Thus far, it is the most comprehensive validation tool for secondary analysis in next generation sequencing.

**Availability and implementation:** Code in Java and Python along with instructions to download the reads and variants is at http://bioinform.github.io/varsim.

**Contact:** rd@bina.com

**Supplementary information:** Supplementary data are available at *Bioinformatics* online.

## 1 Introduction

Due to the lack of ground truth for real data, simulation is a common approach for the evaluation of high-throughput sequencing's secondary analysis, ranging from alignment to variant calling. An early attempt to perform validation without simulation is given in Zook *et* *al.* (2014). However, their attempt involved extensive biological experiments and does not cover the full spectrum of variants. We present the first integrated pipeline that provides complete validation of secondary analysis through simulation as well as analysis with real data.

Most tools simulate variants, but no single tool simulates the full spectrum of variants from small variants to all types of structural variations (SVs). RSVSim (Bartenhagen and Dugas, 2013) simulates SVs, but does not simulate SNVs and small indels. It also does not generate reads. SMASH (Talwalkar *et* *al.*, 2014) only considers SV deletions and insertions. Other variant simulation tools exist (see Supplementary Material); however, VarSim is the only one able to simulate SNVs, small indels and many types of SVs. This completeness allows VarSim to be closely representative of real sequencing studies.

Furthermore, among the aforementioned tools, only a few simulate both variants and reads. VarSim goes further with the ability to validate the correctness of read alignments even near complex SVs.

## 2 Methods

**Fig. 1.**
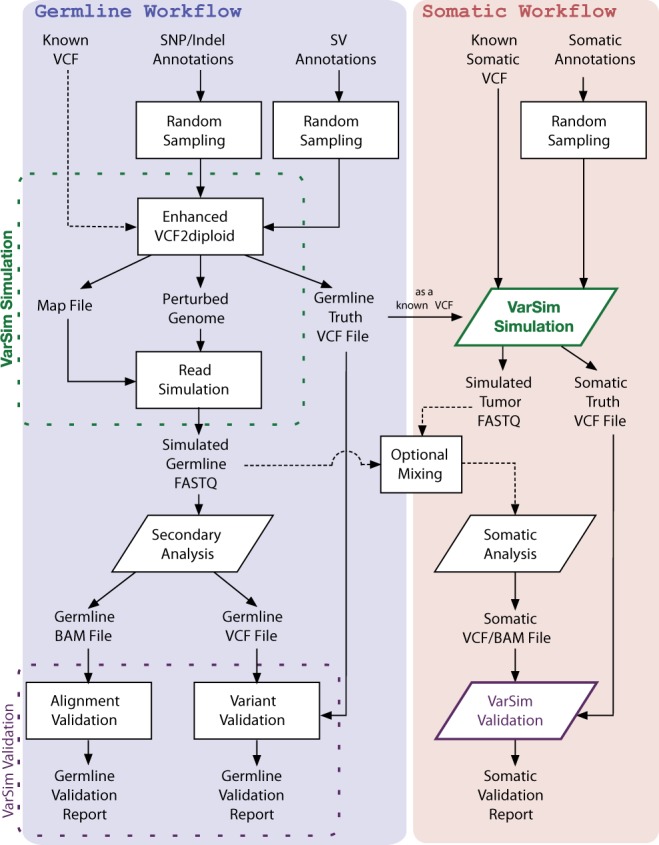
VarSim simulation and validation workflow. The germline workflow can be run with or without the somatic workflow

VarSim works in two steps. The first step is simulation. A perturbed diploid genome is generated by inserting variants into a user-provided reference genome (e.g. GRCh37). Reads are then simulated from this perturbed genome. These reads are processed using the secondary analysis pipeline under consideration [e.g. BWA + GATK (Lam *et* *al.*, 2012)]. The second step is validation. The aligned reads and called variants are validated against the true alignments and variants, respectively. Following that, our reporting tools generate detailed interactive plots showing the accuracy of alignment and variant calling. It is also possible to compare the accuracy between multiple tools. Figure 1 provides an overview of the basic germline workflow.

The basic workflow can also be adapted for simulation of tumor/normal pairs and the validation of somatic variant callers (Fig. 1). VarSim is run twice, once with somatic variants from the COSMIC (Forbes *et* *al.*, 2014) database and/or a somatic variant VCF, and once without any somatic variants. The two sets of reads generated can be optionally mixed to simulate normal contamination at various allele frequencies. After somatic variant analysis is run on the two sets of reads, somatic variants can then be validated in the same way as in the standard germline workflow. See the Supplementary Material for more details.

### 2.1 Simulation

For generating a perturbed genome, VarSim samples small variants and SVs from existing databases (e.g. dbSNP, DGV) and/or a provided VCF file. For SV insertions without a known novel sequence, VarSim generates a new insertion sequence from a database of known human insertion sequence (e.g. the Venter genome insertion sequences). It then generates a diploid genome containing the sampled variants with an enhanced version of vcf2diploid (Rozowsky *et* *al.*, 2011) (see Supplementary Material). Specifically, we added support for handling more types of SVs (inversions, duplications) and improved VCF reading. We also added the ability to generate a map file (MFF, see Supplementary Material) between the perturbed genome and the reference genome. This map is used to convert locations on the perturbed genome to locations on the reference genome. It is more flexible than the chain file in the original vcf2diploid as it can handle complex SVs such as translocations, which will be simulated by VarSim in a future version.

VarSim currently supports DWGSIM and ART (Huang *et* *al.*, 2012). It uses ART as the default since ART learns an error profile based on real sequencing reads. VarSim is flexibly designed to support any type of read simulator with minimal work, this is important because unlike the structure of the human genome, sequencing technology will continue to evolve and change.

As the reads are generated from the perturbed genome, the true alignment location on the reference genome is not available. To determine the true alignment location on the reference genome, VarSim utilizes the map file generated in the genome simulation step. In addition, VarSim parallelizes the read generation of any read simulator to greatly reduce simulation time.

### 2.2 Validation

VarSim validates alignments via meta-data stored in the read name. All possible true read alignment locations are stored in the meta-data. This allows VarSim to validate alignments overlapping the breakpoints of SVs. Furthermore, each alignment is annotated with the type of region it was generated from, which allows validating only the alignments overlapping specific types of variants. An alignment is called correct if it is close to any of the true locations. For instance, if a read overlaps the edge of an inversion, the read could either be aligned partially outside the inversion with the rest soft-clipped or partially inside the inversion and similarly soft-clipped. VarSim validates against all of these possible alignments.

VarSim validates variants by comparing them to the true set of variants inserted into the perturbed reference genome. VarSim handles the variety of possible encodings for a VCF record by normalizing each record to a canonical form before comparison. The accuracy of variant calling is reported based on sensitivity (TPR) and precision (PPV). VarSim reports TPR and PPV broken down into bins by variant type and also variant size. For details of the computation, please see Supplementary methods.

### 2.3 Analysis output

The resulting analysis output for alignment and variant validation is a JSON file that can be visually analyzed as a single interactive HTML document with SVG plots. The plots are generated using the D3 library. Validation metrics include sensitivity, precision and F1 score, which is the harmonic mean of precision and sensitivity (see Supplementary methods). The HTML document is also able to compare multiple analysis outputs. This platform agnostic format makes sharing and comparing results relatively simple.

## 3 Results

**Fig. 2.**
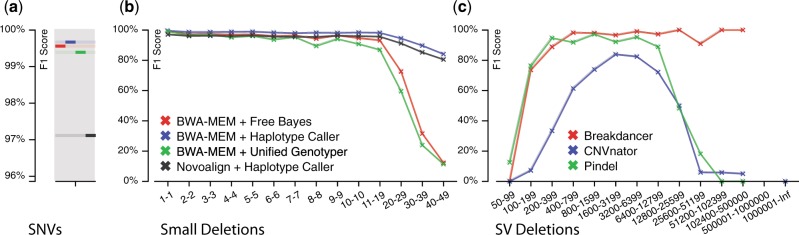
Validation results for some popular secondary analysis tools

We demonstrated VarSim’s completeness in both simulation and validation by simulating NA12878’s personal genome with small variants from genome in a bottle (GiaB) high-confidence regions (Zook *et* *al.*, 2014), and with SVs from 1000 genomes (Mills *et* *al.*, 2011) and DGV (MacDonald *et* *al.*, 2014). Reads were generated at 50× coverage. The accuracy on the simulated reads was similar to the accuracy from the Illumina platinum genome reads of NA12878 (see Supplementary methods). Figure 2a and b present some benchmarking results on SNVs and small deletions. For all variant calling comparisons we used the alignments from BWA-MEM (Li, 2013) after realignment and recalibration with GATK unless otherwise specified. Novoalign’s alignments were used directly as input to Haplotype Caller without realignment and recalibration as recommended by the authors. In this case, Novoalign performed slightly worse in comparison to BWA-MEM. For small deletions, Haplotype Caller performed the best when compared to both Unified Genotyper (McKenna *et* *al.*, 2010) and FreeBayes (Garrison and Marth, 2012), especially for larger deletions. The results on SV deletions from several popular SV calling tools (Abyzov *et* *al.*, 2011; Chen *et* *al.*, 2009; Ye *et* *al.*, 2009) are shown in Figure 2c. The three tools represented three different methods for SV calling—split-read, read-depth and paired-end. All tools performed well for moderate-sized deletion SVs. Only BreakDancer (paired-end mapping) was able to recover larger SV deletions. However, it was not able to recover exact breakpoints. All tools failed to adequately recover deletion SVs in the smaller range. When comparing somatic analysis tools MuTect (Cibulskis *et* *al.*, 2013) was superior to the other tools, especially when the tumor allele frequency was low. Additional analysis of secondary and somatic analysis tools based on the simulated NA12878 genome are provided in the Supplementary Material.

## 4 Conclusions and future work

VarSim is the most comprehensive pipeline for simulation and validation of secondary analysis, covering both small variants and SVs on a diploid genome. Future work on VarSim will add support for translocations, as well as interspersed duplications.

We envision VarSim will become an invaluable tool in the evaluation of new secondary analysis methods.

## Supplementary Material

Supplementary Data
