# Supplementary material for: VarSim: a high-fidelity simulation and validation framework for high-throughput genome sequencing with cancer applications
Source: Bioinformatics. 2014 Dec 17;31(9):1469–71. doi: 10.1093/bioinformatics/btu828 (PMC4410653; doi:10.1093/bioinformatics/btu828)
Supplement: Supplementary Data [file supp_31_9_1469__index.html]

VarSim: A high-fidelity simulation and validation framework for high-throughput genome sequencing with cancer applications — VarSim: a high-fidelity simulation and validation framework for high-throughput genome sequencing with cancer applications — VarSim: a high-fidelity simulation and validation framework for high-throughput genome sequencing with cancer applications — Supplementary Data 

# VarSim: a high-fidelity simulation and validation framework for high-throughput genome sequencing with cancer applications

## Supplementary Data

files

**Files in this Data Supplement:**

- Supplementary Data - pdf file
