# Supplementary material for: VarSim: a high-fidelity simulation and validation framework for high-throughput genome sequencing with cancer applications
Source: Bioinformatics. 2014 Dec 17;31(9):1469–71. doi: 10.1093/bioinformatics/btu828 (PMC4410653; doi:10.1093/bioinformatics/btu828)
Supplement: Supplementary Data [file supp_btu828_supp.pdf]

VarSim: A high-fidelity simulation and validation framework for high-throughput genome sequencing with cancer applications

SUPPLEMENTARY INFORMATION

John C. Mu*, Marghoob Mohiyuddin *, Jian Li,
Narges Bani Asadi, Mark B. Gerstein, Alexej Abyzov,
Wing H. Wong and Hugo Y.K. Lam†

December 2, 2014

1 Pre-generated Data

For the convenience of researchers we generate to 100x coverage the following three genomes and made the following data available online.

- **Female personal genome:** We used high-confidence variants for NA12878 from literature (Abecasis *et al.*, 2010; Mills *et al.*, 2011) and used read simulation to generate the reads.
- **Male personal genome:** Similar to the above, except that we used variants from NS12911 (the Venter genome (Pang *et al.*, 2010; Levy *et al.*, 2007)). This includes exact insertion breakpoints and novel insertion sequences.
- **Female tumor genome:** A random subset of variants from the COSMIC (Danecek *et al.*, 2011) database was added to the "Female personal genome". Normal contamination was simulated at 0.1 somatic allele frequency.

*The authors wish it to be known that, in their opinion, the first two authors should be regarded as joint First Authors.
†To whom correspondence should be addressed.

2 Other Existing Simulation Tools

VarSim currently supports DWGSIM, and ART (Huang *et al.*, 2012). They are the two most common and popular read simulators. DWGSIM generates base error qualities based on a parametric model. ART attempts to learn the quality score distribution from read sequencer reads. Both these tools have the limitation that they simulate sequencing errors based on the base quality reported. Hence, base quality re-calibration is not required.

Other read simulation tools include GemSIM (McElroy *et al.*, 2012) and pIRS (Hu *et al.*, 2012) that generate detailed error profiles based on aligned reads; this mitigates the need to trust the based qualities generated by the sequencer. If the alignment is correct, this will generate more realistic base qualities. Wessim (Kim *et al.*, 2013) is specifically designed for simulating exome sequencing reads. PBSIM (Ono *et al.*, 2013) is designed for Pacific Biosciences™ reads.

Other SV simulation tools (Xi *et al.*, 2011; Zhang *et al.*, 2011; Mimori *et al.*, 2013) exist as part of SV callers and are also not comprehensive.

3 Supplementary Methods

This section described in detail the methods used by VarSim for both simulation and validation.

3.1 Simulation

Simulation involves first generating a perturbed diploid genome with the desired variants. Reads are then simulated from this perturbed genome.

3.1.1 Genome

The set of variants that are inserted into the reference genome to create the perturbed genome must be representative of the amount of and type of variation observed in a typical individual. VarSim simulates SNVs, deletions, insertions, MNPs, complex variants, tandem duplications and inversions. The number of each type of variant is specified by the user. The distribution of the variant size within each type is the empirical distribution in the provided database. For a human reference we use dbSNP build 138 (Sherry *et al.*, 2001) for variants less than 50 bp and DGV (MacDonald *et al.*, 2014) for variants larger than or equal to 50 bp. The contents of an insertion with unknown novel sequence are randomly sampled from a user provided insertion sequence file. For a human reference, we use the concatenation of Venter insertion sequences (Levy *et al.*, 2007). Novel variants are generated by randomly choosing a variant in the database and randomly moving it to a new location within the same chromosome. This preserves the size distribution of the variants as well as the distribution of variants among chromosomes. The phase of variants is randomly assigned if not provided.

In order to generate a diploid genome with the simulated variants we enhanced vcf2diploid (Rozowsky *et al.*, 2011) for this purpose. Specifically, we added support for handling more types of SVs (inversions, duplications) and improved VCF reading. We also added the ability to generate a map between the perturbed genome and the reference genome in the new map file format (see Section 3.1.1). This map is used to convert locations on the perturbed genome to locations on the reference genome. It is more flexible than the traditional chain file in the original vcf2diploid since it can easily handle complex structural variants such as translocations, which will be simulated by VarSim in a future version.

Map file format (MFF) The map file format contains 8 columns:

```
<size_of_block> <host_chr> <host_loc>
<ref_chr> <ref_loc> <direction_of_block>
<feature_name> <variant_id>
```

It records a map between blocks of the perturbed genome to blocks of the reference genome and vice versa. Each line describes the mapping of one block. The direction of the block (+ or -) indicates whether the block is reverse complemented. The feature name indicates the type feature a block comes from; `INS`, `DEL`, `DUP`, `DUP_TANDEM`, `SEQ` or `INV`. When the feature is `INS`, the `ref_loc` field indicates the location before which the insertion occurred. This is vice versa for `DEL`. `variant_id` is used to keep track of variants that built from multiple blocks. Also, all locations are 1-based.

The MFF can be thought of as a compression of the naive method of providing mapping for each individual location in the perturbed genome — the compression groups together consecutive locations which map to consecutive locations or reversed consecutive locations. In addition, the `feature_name` annotation helps to identify which variation the block is part of, which can be helpful in validation. In the case of inversions, the direction of the block would be "-" to indicate the sequence in the perturbed genome block is reversed with respect to the reference genome block. We note that in the case of blocks corresponding to an inserted sequence, there is no corresponding block on the reference genome — in this case, we report the location in the reference before the inserted sequence. Similarly, in the case of deletions, we indicate the location in the perturbed genome after which the deletion happened. Thus, the MFF allows conversion of locations between the perturbed and the reference genomes. In VarSim, we use it for converting alignments from the perturbed genome to the reference genome.

3.1.2 Reads

VarSim calls external tools to perform read simulation. This allows flexibility in supporting future sequencing platforms. Since the reads are generated from the perturbed genome, the true alignment location on the reference genome is not

available. In order to determine the true alignment location on the reference genome, VarSim utilizes the MFF generated in the genome simulation step.

Currently, VarSim supports ART (Huang *et al.*, 2012) and DWGSIM (Homer, 2014) for read simulation and either of them can be used to simulate the reads. ART is the default choice since it is well established and supports simulating from a sequencer error profile learned from real data. Since read simulation is slow (compute-intensive single-threaded code), it is a significant bottleneck for any simulation validation tool. In order to improve the speed of read simulation, we generate multiple sets of FASTQs in parallel by leveraging modern multi-core CPUs—since these tools are typically limited by CPU performance, generating multiple FASTQs in parallel speeds up read simulation almost linearly. Furthermore, these tools typically require a seed for the random-number generator, we are careful to generate the different FASTQs with different seeds to ensure that the FASTQs are not identical when generating multiple FASTQs. Once the simulated FASTQs have been generated, they are lifted over to the reference genome (e.g. GRCh37) by updating the source locations in the read names. This meta-data is stored in the read names for simplicity of validation as the reads are permuted from their original location in the file after alignment and sorting. If a read spans multiple map blocks then the lift-over can yield multiple locations along with different orientations—this means that alignment validation would match against all the possible source locations and orientations. In order to save disk space, all FASTQs are compressed when written to disk. Furthermore, to avoid any performance hit due to compression, the compression/decompression is run in parallel with read simulation and lift-over.

The lift-over requires each read to be annotated with the locations where the read was simulated from in the read name. Hence, a simple parser is needed for each read simulator. We have built parsers for both ART and DWGSIM.

3.2 Validation

Two types of validation are possible with VarSim. Firstly, the alignment of the reads can be validated with the true alignment locations. Secondly, the called variants can be compared to the true variants. It is useful to validate the read alignments as well as the called variants since incorrectly called variants are frequently related to mis-alignments. VarSim is also able to accept a BED file (Quinlan and Hall, 2010) as input and only perform validation within the BED file specified regions. This is useful for focused studies only interested in specific genes or genomic regions.

3.2.1 Read Alignments

VarSim validates alignments via meta-data stored in the read name of the SAM/BAM (Li *et al.*, 2009) file, which characterizes the true alignment location. As described in Section 3.1.2, all possible true read alignment locations are reported in the read meta-data. This allows VarSim to validate alignments overlapping the breakpoints of structural variants. An alignment is called cor-

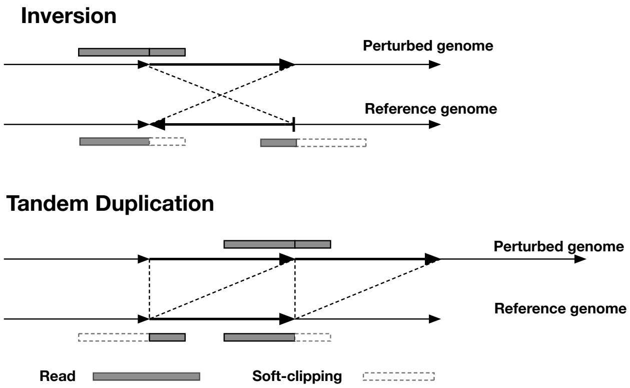

Figure 1: Structural variants can cause reads to have multiple possible alignments

rect if it is close to any of the true locations (see Figure 1). For instance, if a read overlaps the edge of an inversion, the read could either be aligned partially outside the inversion with the rest soft-clipped or partially inside the inversion and similarly soft-clipped. VarSim validates against all of these possible alignments. The wiggle parameter that defines closeness can be set by the user.

The accuracy of read alignment is reported as a plot of true positive rate (TPR) versus false discovery rate (FDR) varying cutoffs of the mapping quality (MAPQ) (Li *et al.*, 2008) score. The area under this curve is also reported.

Furthermore, each read is annotated with the type of region it was generated from so during the validation it is possible to examine only the reads overlapping insertions, deletions or any other type of variant. The provides detailed accuracy reports on reads overlapping each type of variant, ignoring the reads without variation.

3.2.2 Called Variants

VarSim validates variants by comparing them to the true set of variants inserted into the perturbed reference genome. The main issue when comparing variants is the definition of a correctly called variant. Due to the flexibility allowed in the VCF format, it is entirely possible for two different variant callers to encode a variant in different ways in a VCF file. It is also possible for variant callers to output different numbers of VCF records for the same group of variants. Hence, all VCF files need to be normalized before comparison. A normalization procedure was also proposed in (Zook *et al.*, 2014). However, their approach is not compatible with reporting methodology that divides the comparison into multiple variant types.

VarSim handles the variety of possible encodings for a VCF record by nor-

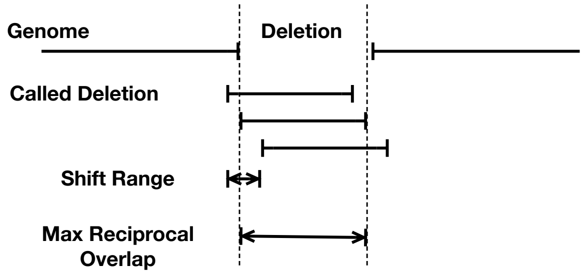

Figure 2: Validation of variants uses this range

malizing each record to a canonical form before comparison. The canonical form is generated by converting the VCF record into a simple insertion or deletion followed by a number of SNVs. VarSim tries to put the insertion or deletion at both the start and the end of the variant. It keeps the representation that results in the least number of mismatches with respect to the reference. This normalization procedure covers most possible variations in encoding. One notable exception is when multiple insertions or deletions are encoded in a single VCF record. The only way to handle this case is to rebuild the entire variation sequence and compare at a sequence level. This sequence level comparison will have to take into account phasing ambiguity. Since this case was only very rarely observed in the tools we examined, this modification is left for future work.

Variant comparison is performed at the allele level. Hence, if genotype concordance is required to be computed, homozygous variants will only match homozygous variants, similarly for heterozygous variants.

The accuracy of variant calling is reported as an F1 score, which is the harmonic mean of sensitivity (TPR) and precision (PPV). The accuracy computation is governed by two parameters γ and δ that represent the overlap ratio and wiggle. A match between two canonical variants is defined between SNVs, insertions and deletions since these are the only types of canonical variants.

SNV: An SNV must be in exactly the same position as another SNV to be called a match. SNVs cannot match insertions or deletions.

Deletion: A deletion's start position can be shifted within the allowable wiggle δ. If the reciprocal overlap is greater than or equal to γ for any shift, it is called a match (see Figure 2).

Insertion: The same as for deletions, but the reference and alternate sequence are inverted.

Computing TPR requires the number of true positives (TP). A TP is defined in terms of the true variants (i.e. variants in the truth set). Let V be an arbitrary true variant, v_i be the canonical variants V is broken down into and $|v_i|$ as the length of the variant. Define the match ratio as

$$\texttt{matchratio} = \frac{\sum_i |v_i| \mathbb{1}_{\{v_i \text{ matches a call}\}}}{\sum_i |v_i|}.$$

If matchratio is greater than or equal to γ, the true variant is called a TP. The TPR is computed as the number of TP (from true variants) divided by the number of true variants.

PPV also requires a count of TP; however, it is computed based on the called variants. If a called variant has a match ratio greater than or equal to γ, then it is marked as a TP. The PPV is computed as the number of TP (from called variants) divided by the number of called variants.

VarSim's computation of TPR and PPV allows it to report accuracy broken down into variant types and also variant size ranges. Intuitively, TPR represents the ability to recover true variation and PPV represents the ability to make less incorrect calls. This is why TPR is computed based on the true variants and PPV is computed based on the variant calls.

The genotype of a variant is compared to the truth by individually considering each allele. For SNVs, both alleles must match exactly for the genotype to be called correct. For insertions and deletions, we only check if the variant is correctly heterozygous or homozygous. The contents does not have to match exactly.

3.2.3 Analysis Output

VarSim outputs the TPR and PPV/FDR for both read alignments and variants. In both cases, the results are grouped by the type of variant. We define variant classes in the following way.

- **Reference**: Identical sequence to the reference
- **SNV**: Length one with different sequence to the reference.
- **Insertion**: Length greater than or equal to one added to reference.
- **Deletion**: Length greater than or equal to one removed from reference.
- **Complex**: All other types of variants, including MNPs
- **Inversion**: Inversion structural variation
- **Tandem Duplication**: Tandem duplication structural variation

For heterozygous variants, we classify variants with alleles from two different classes as "Complex".

For read alignments, the output is grouped by the type of variant the read overlaps in the truth set and also plotted for a range of mapping quality scores (Li *et al.*, 2008).

For variants, the output is grouped by the type of variant and also length of variant. For heterozygous variants, the length is given as the maximum length

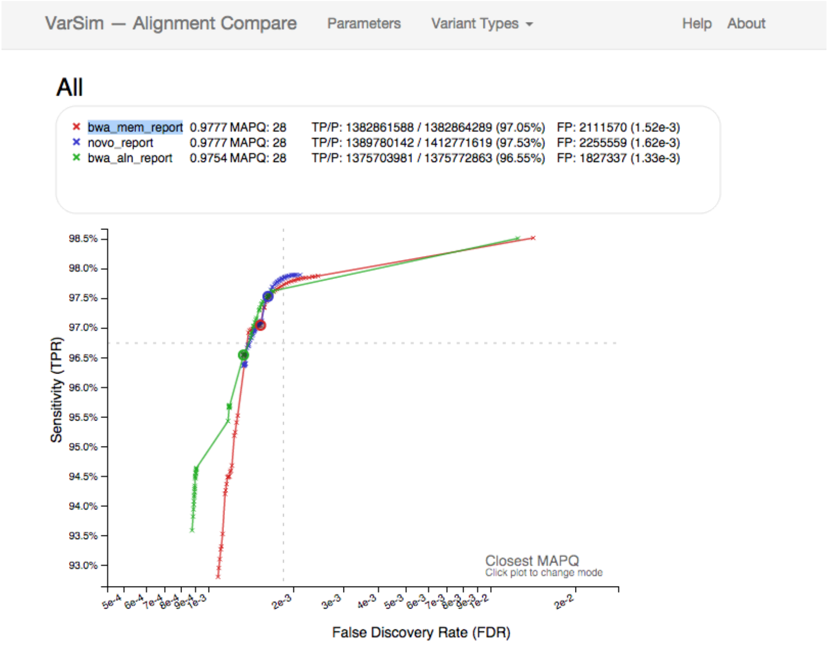

Figure 3: Cropped view of the alignment comparison page in a web browser

of all alleles. For both cases an optional BED file can be provided to restrict the analysis to specific regions.

The resulting analysis output is a JSON file that can be viewed as a single HTML document with SVG plots generated using the D3 (Bostock *et al.*, 2011) library. This platform agnostic format makes sharing and comparing results relatively simple.

Figures 3 and 4 show examples of the comparison pages as viewed in a web browser.

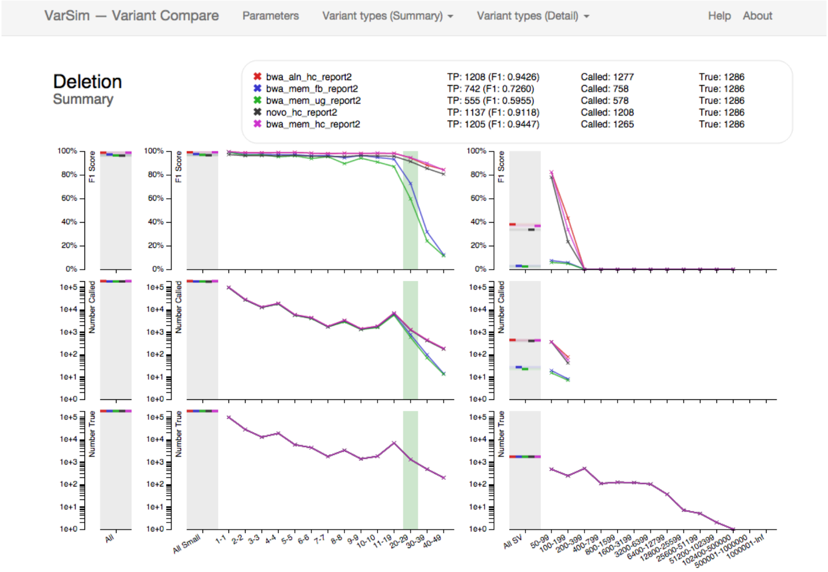

Figure 4: Cropped view of the variant comparison page in a web browser

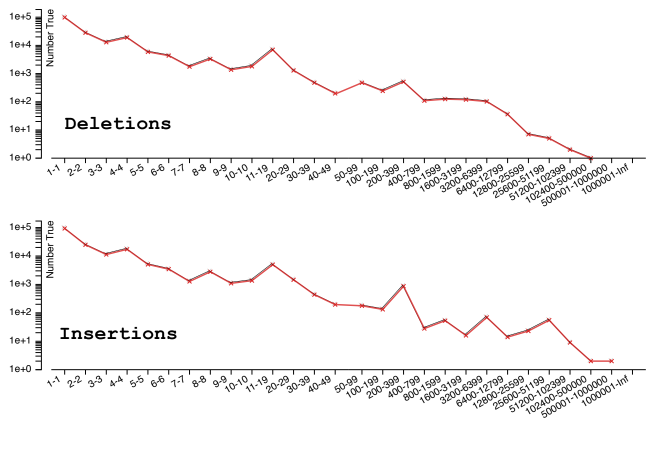

Figure 5: Histogram of insertions and deletions lengths simulated

4 Supplementary Results

In order to demonstrate VarSim's completeness in both simulation and validation, we simulated NA12878's personal genome with variants from genome in a bottle (GiaB) high-confidence regions (Zook *et al.*, 2014) with structural variations from 1000 Genomes (Mills *et al.*, 2011) and DGV (MacDonald *et al.*, 2014) and generated reads to 50x coverage. We used the high-confidence regions for this comparison since it should be a baseline where all tools are expected to perform well. This personal genome is used to compare the performance of several common secondary analysis tools. The distribution of the indel sizes are provided in Figure 5.

The aligners we considered for this analysis were BWA-backtrack (Li and Durbin, 2009), BWA-MEM (Li, 2013) and Novoalign (Novocraft Technologies, 2014). Novoalign is run without GATK realignment and GATK base recalibration as recommended by authors. The small variant calling algorithms we considered were HaplotypeCaller (HC) and UnifiedGenotyper (UG) from the Genome Analysis Toolkit (McKenna *et al.*, 2010), and FreeBayes (FB) (Garrison and Marth, 2012). UG and HC were run following best practices from the Broad Institute (Van der Auwera *et al.*, 2013) while FB was run with the default settings. For structural variation calling we used Pindel (Ye *et al.*, 2009), CNVnator (Abyzov *et al.*, 2011) and BreakDancer (Chen *et al.*, 2009). We also analyzed the simulated tumor genome with VarSim. The somatic variant callers MuTect (Cibulskis *et al.*, 2013), VarScan2 (Koboldt *et al.*, 2012), JointSNVMix (Roth *et al.*, 2012) and Somatic Sniper (Larson *et al.*, 2011) were compared based on this simulated genome.

Overall, the following results only represent a subset of what VarSim outputs. The reader is encouraged to explore further at the VarSim website, where all of

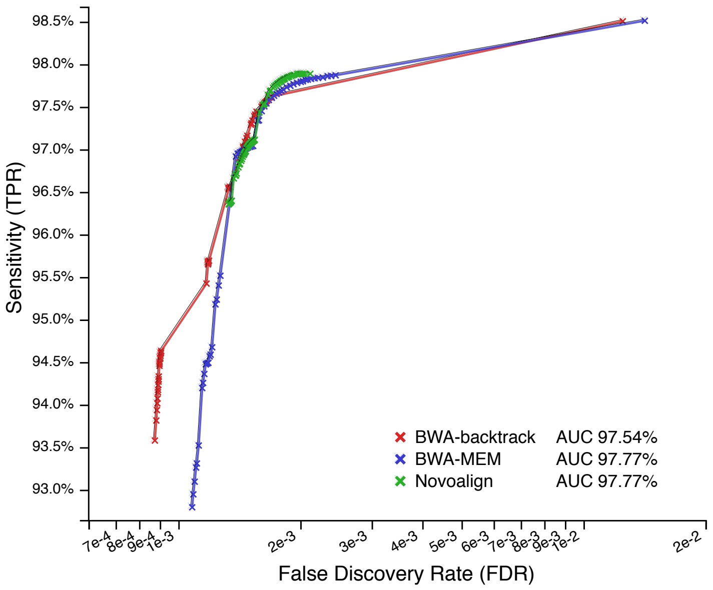

Figure 6: Alignment accuracy of all reads

the datasets for the following results are available.

4.1 Alignment Accuracy

VarSim was used to compare the alignment accuracy of BWA-backtrack, BWA-MEM and Novoalign before realignment. The results are shown in Figure 6. Overall they all performed very well on the 100 bp paired-end reads. Novoalign and BWA-MEM were slightly more accurate compared to BWA-backtrack in terms of area under the curve[1] (AUC). However, BWA-backtrack is able to achieve a lower error floor.

4.2 Variant Calling Accuracy

For all variant calling comparisons we used the results of BWA-MEM after re-alignment and re-calibration with GATK. Figures 7 and 8 show the accuracy for simple small indels. We used 20 bp wiggle and 80% reciprocal overlap as the matching criteria. The F1 score, which is the harmonic mean of precision and sensitivity (Section 3.2.2), is reported as a measure of accuracy. HaplotypeCaller performs very well and was superior to both UnifiedGenotyper and FreeBayes,

[1]This definition of AUC is based on TPR and FDR rather than the traditional TPR and FPR. It should only be used as a guide as TPR vs FDR is not guaranteed to be monotonically increasing.

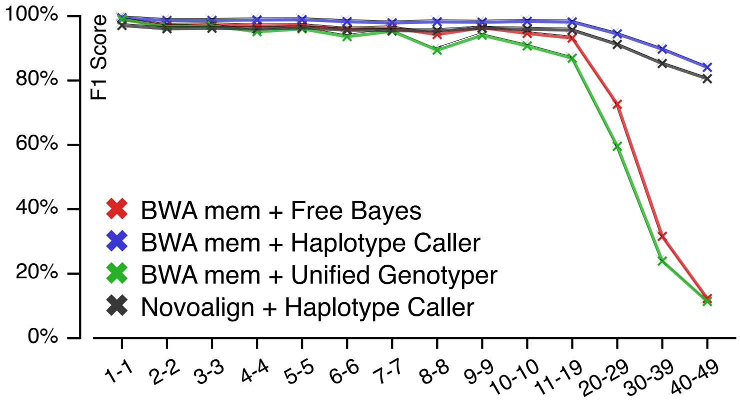

Figure 7: Small deletion accuracy

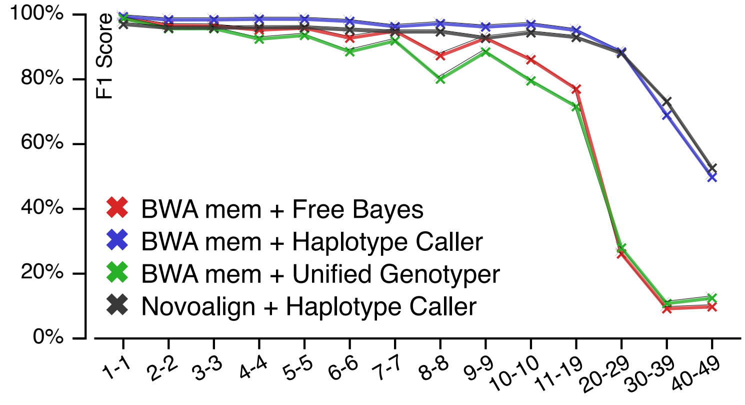

Figure 8: Small insertion accuracy

especially for larger indels. However, we note that all callers suffered a loss in accuracy for indels greater than 10 bp.

We also compared the effect of using different aligners on the accuracy of variant calling. In particular, we compared Novoalign and BWA-MEM as input to Haplotype Caller. BWA-MEM was run with GATK realignment and GATK base quality calibration, while Novoalign was run without as recommended by the authors. We found a slight, but significant difference in the resulting variant calling accuracy. For SNVs, the F1 score was 0.997 for BWA-MEM and 0.971 for Novoalign. For deletions, the F1 score was 0.986 for BWA-MEM and 0.961 for Novoalign. For insertions, the F1 score was 0.980 for BWA-MEM and 0.955 for Novoalign. We believe this difference could be attributed to realignment. However, this would require further study.

Figure 9 shows the results of some popular structural variation callers on

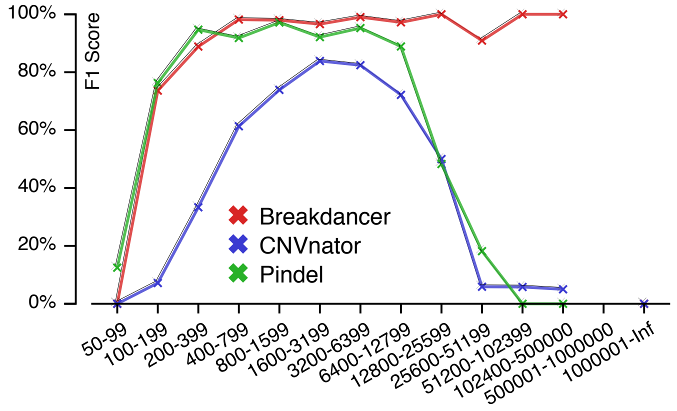

Figure 9: SV deletion accuracy

large deletions. We used 100 bp wiggle and 50% reciprocal overlap as the matching criteria. The three tools represented three different methods for SV calling – Split-read, read-depth and paired-end. All tools performed well for moderate-sized deletion SVs. Only BreakDancer was able to recover larger deletion SVs. However, it was not able to recover exact breakpoints. All tools failed to adequately recover deletion SVs in the smaller range. Insertion SVs are much harder to recover via short reads with limited insert size. No tools were able to recover insertion SVs beyond 200 bp. For insertion SVs less than 200 bp, breakdancer recovered a small number of them.

4.3 Somatic Variant Calling Accuracy

Accuracy results for somatic SNV variation calling at two different allele frequencies is shown in Figure 10. We used a pure normal sample for this analysis. Overall, MuTect was superior to the other tools. When the tumor allele frequency was 0.1, the difference was much more stark. Only MuTect and VarScan2 were able to call somatic indels. At 0.3 allele frequency, the F1 score for insertions was 0.41 for MuTect and 0.43 for VarScan2. For deletions the F1 score was 0.42 for MuTect and 0.50 for VarScan2. Overall, VarScan2 had a higher sensitivity at the cost of lower precision, while MuTect had lower sensitivity and higher precision. At 0.1 allele frequency, both tools essentially found no somatic indel mutations.

4.4 Real Data Comparison

NA12878 is a well studied individual and hence an abundance of real sequencing data is available. One such set is the Illumina platinum genome (IPG) sequence of NA12878, sequenced to 50x coverage (ERP001960). We compared

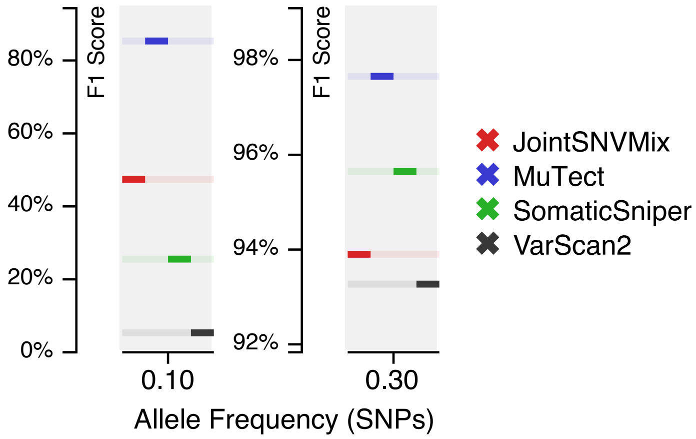

Figure 10: Somatic SNV calling accuracy at two allele frequencies

the accuracy of variants called from this set to the accuracy of the calls from the VarSim simulated reads. BWA-MEM was used for alignment. Haplotype Caller and FreeBayes was used for variant calling. Figures 11 and 12 show the results for insertions and deletions. For SNVs, the overall F1 score was 0.9967 for VarSim and 0.9950 for IPG. Overall, the F1 scores were close. We found that the differences in insertions and deletions were mostly due to limitations in the read simulator. In particular, ART does not account for the low quality bases typically found around homopolymers for Illumina reads.

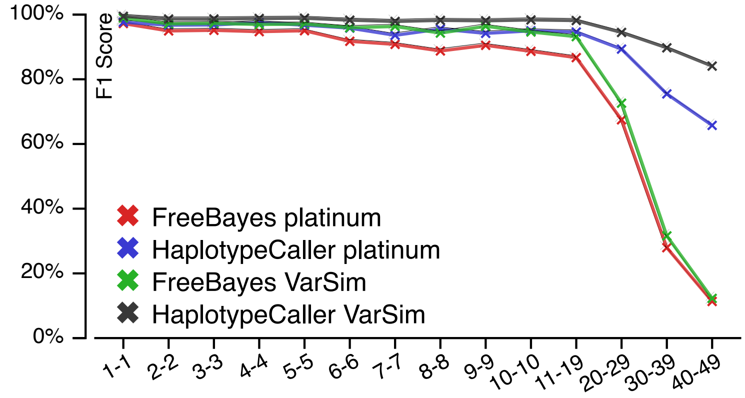

Figure 11: Comparison with platinum genome for deletion calling accuracy

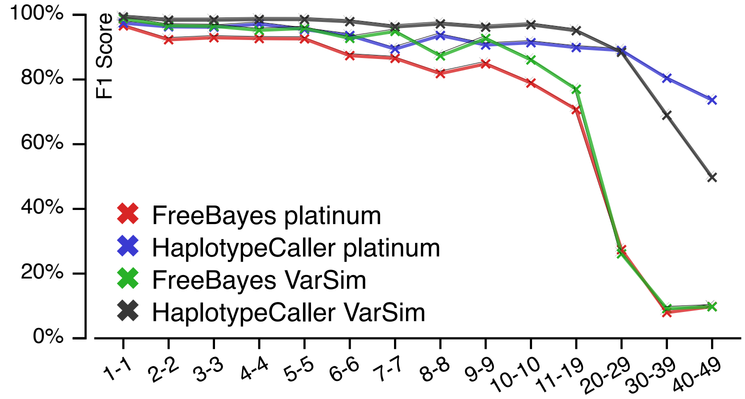

Figure 12: Comparison with platinum genome for insertion calling accuracy

References

Abecasis, G.R. et al (2010). A map of human genome variation from population-scale sequencing. *Nature*, **467**(7319), 1061–1073.

Abyzov, A., Urban, A.E., Snyder, M. and Gerstein, M. (2011). Cnvnator: An approach to discover, genotype, and characterize typical and atypical cnvs from family and population genome sequencing. *Genome Research*, **21**(6), 974–984.

Bostock, M., Ogievetsky, V. and Heer, J. (2011). D3: Data-Driven Documents. *IEEE Transactions on Visualization and Computer Graphics*, **17**(12), 2301–2309.

Chen, K. et al (2009). BreakDancer: an algorithm for high-resolution mapping of genomic structural variation. *Nat. Methods*, **6**(9), 677–681.

Cibulskis, K. et al (2013). Sensitive detection of somatic point mutations in impure and heterogeneous cancer samples. *Nat Biotechnol*, **31**(3), 213–219.

Danecek, P. et al (2011). The variant call format and VCFtools. *Bioinformatics*, **27**(15), 2156–2158.

Garrison, E. and Marth, G. (2012). Haplotype-based variant detection from short-read sequencing. *arXiv preprint arXiv:1207.3907 [q-bio.GN]*.

Homer, N. (2014). Whole Genome Simulation.

Hu, X. et al (2012). pIRS: Profile-based Illumina pair-end reads simulator. *Bioinformatics*, **28**(11), 1533–1535.

Huang, W., Li, L., Myers, J.R. and Marth, G.T. (2012). ART: a next-generation sequencing read simulator. *Bioinformatics*, **28**(4), 593–594.

Kim, S., Jeong, K. and Bafna, V. (2013). Wessim: a whole-exome sequencing simulator based on in silico exome capture. *Bioinformatics*, **29**(8), 1076–1077.

Koboldt, D.C. et al (2012). VarScan 2: Somatic mutation and copy number alteration discovery in cancer by exome sequencing. *Genome Research*, **22**(3), 568–576.

Larson, D.E. et al (2011). SomaticSniper: identification of somatic point mutations in whole genome sequencing data. *Bioinformatics*, **28**(3), 311–317.

Levy, S. et al (2007). The diploid genome sequence of an individual human. *PLoS Biol.*, **5**(10), e254.

Li, H. (2013). Aligning sequence reads, clone sequences and assembly contigs with BWA-MEM. *arXiv*, **arXiv:1303.3997 [q-bio.GN]**.

Li, H. and Durbin, R. (2009). Fast and accurate short read alignment with burrowswheeler transform. *Bioinformatics*, **25**(14), 1754–1760.

Li, H., Ruan, J. and Durbin, R. (2008). Mapping short DNA sequencing reads and calling variants using mapping quality scores. *Genome Res.*, **18**(11), 1851–1858.

Li, H. et al (2009). The Sequence Alignment/Map format and SAMtools. *Bioinformatics*, **25**(16), 2078–2079.

MacDonald, J.R., Ziman, R., Yuen, R.K.C., Feuk, L. and Scherer, S.W. (2014). The Database of Genomic Variants: a curated collection of structural variation in the human genome. *Nucleic Acids Research*, **42**(D1), D986–D992.

McElroy, K., Luciani, F. and Thomas, T. (2012). GemSIM: general, error-model based simulator of next-generation sequencing data. *BMC Genomics*, **13**(1), 74.

McKenna, A. et al (2010). The genome analysis toolkit: A MapReduceframework for analyzing next-generation DNAsequencing data. *Genome Research*, **20**(9), 1297–1303.

Mills, R.E. et al (2011). Mapping copy number variation by population-scale genome sequencing. *Nature*, **470**(7332), 59–65.

Mimori, T. et al (2013). iSVP: an integrated structural variant calling pipeline from high-throughput sequencing data. *BMC Systems Biology*, **7**(6), 1–8.

Novocraft Technologies (2014). Novoalign.

Ono, Y., Asai, K. and Hamada, M. (2013). PBSIM: PacBio reads simulatortoward accurate genome assembly. *Bioinformatics*, **29**(1), 119–121.

Pang, A.W. et al (2010). Towards a comprehensive structural variation map of an individual human genome. *Genome Biol.*, **11**(5), R52.

Quinlan, A.R. and Hall, I.M. (2010). Bedtools: a flexible suite of utilities for comparing genomic features. *Bioinformatics*, **26**(6), 841–842.

Roth, A. et al (2012). JointSNVMix: a probabilistic model for accurate detection of somatic mutations in normal/tumour paired next-generation sequencing data. *Bioinformatics*, **28**(7), 907–913.

Rozowsky, J. et al (2011). AlleleSeq: analysis of allele-specific expression and binding in a network framework. *Molecular Systems Biology*, **7**(1).

Sherry, S.T. et al (2001). dbSNP: the NCBI database of genetic variation. *Nucleic Acids Research*, **29**(1), 308–311.

Van der Auwera, G.A. et al (2013). *From FastQ Data to High-Confidence Variant Calls: The Genome Analysis Toolkit Best Practices Pipeline*, chapter 11. John Wiley & Sons, Inc.

Xi, R. et al (2011). Copy number variation detection in whole-genome sequencing data using the Bayesian information criterion. *Proceedings of the National Academy of Sciences*, **108**(46), E1128–E1136.

Ye, K., Schulz, M.H., Long, Q., Apweiler, R. and Ning, Z. (2009). Pindel: a pattern growth approach to detect break points of large deletions and medium sized insertions from paired-end short reads. *Bioinformatics*, **25**(21), 2865–2871.

Zhang, Z. et al (2011). Identification of genomic indels and structural variations using split reads. *BMC Genomics*, **12**(1), 375.

Zook, J.M. et al (2014). Integrating human sequence data sets provides a resource of benchmark SNP and indel genotype calls. *Nat. Biotechnol.*, **32**(3), 246–251.
